# Limited predictive power of known resistance genes for phenotypic drug resistance in clinical *Mycobacterium abscessus* complex from Beijing in China

**DOI:** 10.1128/aac.01847-24

**Published:** 2025-05-27

**Authors:** Yue Hou, Rui Pi, Junnan Jia, Zhaojun Wu, Fengmin Huo, Yu Zhou, Hui Jiang, Howard E. Takiff, Chendi Zhu, Wei Wang, Weimin Li

**Affiliations:** 1 Beijing Chest Hospital, Capital Medical University 12517, Beijing, China; 2 Beijing Tuberculosis and Thoracic Tumor Research Institute 117550 , Beijing, China; 3 Changde Hospital, Xiangya School of Medicine, Central South University (The First People’s Hospital of Changde City) 117718, Changde, Hunan, China; 4 CMBC, Instituto Venezolano de Investigaciones Científicas, IVIC 37590, Caracas, Venezuela; Bill & Melinda Gates Medical Research Institute, Cambridge, Massachusetts, USA

**Keywords:** *Mycobacterium abscessus* complex, whole-genome sequencing (WGS), phenotypic antimicrobial susceptibility testing, genotype

## Abstract

*Mycobacterium abscessus* complex (MABC) is an emerging pathogen with intrinsic multidrug resistance. Genomic sequencing technology has been widely applied to predict bacterial resistance in other bacteria, but the catalog of known resistance-determining genes to explain phenotypic resistance in the MABC is incomplete for many antibiotics. Eighty-one MABC strains were isolated from sputum samples of patients with pulmonary disease in the Beijing Chest Hospital. All isolates were tested for minimum inhibitory concentrations (MICs) to eight antibiotics and underwent whole-genome sequencing (WGS). Of the total 81 MABC isolates, six strains exhibited clarithromycin (CLM) resistance by day 3 in culture, but only one (16.7%, 1/6) contained a mutation in the *rrl* gene. All *M. abscessus* strains contained the *erm (41)28T* (100.0%, 49/49) polymorphism and exhibited CLM-induced resistance after 14 days in culture. Of the 61 imipenem-resistant strains, 12 (19.7%, 12/61) had mutations in the *bla* gene. Although there were four (4.9%) amikacin-resistant, nine (11.1%) linezolid-resistant, eight (9.9%) clofazimine-resistant, 23 (28.4%) bedaquiline-resistant, and 27 (33.3%) cefoxitin-resistant strains, no known mutations associated with resistance to these antibiotics were found. These results suggest that the explanatory power of known resistance genes for clinical MABC resistance is limited and that other unidentified genes or novel resistance mechanisms may be involved.

## INTRODUCTION

The *Mycobacterium abscessus* complex (MABC) comprises three subspecies: *M.abscessus*, *M.massiliense*, and *M.bolletii*, which have become emerging pathogens with a predilection for causing pulmonary infections in patients with chronic lung diseases, damaged lungs, or impaired immune systems (1). The MABC bacteria are inherently multidrug-resistant, and current treatment options are limited. Drug regimens generally include a macrolide (azithromycin [AZM] or clarithromycin [CLM]) and amikacin (AK) in combination with a β-lactam such as imipenem (IPM) or cefoxitin (FOX), and frequently also bedaquiline (BDQ), linezolid (LZD) or clofazimine (CFZ) (2). Intrinsic and acquired drug resistance make treatment of MABC infections difficult for both patients and physicians, highlighting an urgent need for new and innovative therapies (3).

More than 30 MABC genes associated with drug resistance have been identified and validated *in vitro* (4). For the macrolide antibiotics CLM and AZM, which are cornerstones for treating MABC infections (5, 6), there are two genotypic resistance determinants: acquired resistance caused by mutations in the *rrl* gene (23S rRNA) at positions 2270/2271 (*E. coli* numbering 2058/2059) (7) and inducible resistance mediated by the intact *erm (41*) gene with a thymine at position 28 [*erm(41*)28T] (8). Based on BTS and ATS/IDSA guideline recommendations (9), AZM is widely used in clinical practice to treat MAB infections. Although both CLM and AZM are macrolide antibiotics, clinical isolates of MABC exhibit significant differences in their resistance phenotypes to these two drugs (10). Therefore, it is necessary to obtain more data on azithromycin resistance in MAB clinical isolates and the resistance mechanisms involved. AK resistance is primarily associated with substitutions A1408G or C1409T in the *rrs* gene (16S rRNA) (11), but it has also been associated with mutations in the *eis1* (12), *eis2* (13), and *rpsL* (14) genes. Drug efflux pumps can play a role in resistance to multiple drugs (15), and efflux pumps encoded by *MAB_4384* and members of the *MmpL* and *MmpS* gene families have been implicated in resistance to CFZ and BDQ (16, 17). Although the frequency of MABC infections in China has been increasing (18), studies on the correlation between genotypic and phenotypic resistance in Chinese MABC isolates are severely lacking (19).

Whole-genome sequencing (WGS) technology is often used for predicting drug resistance in *M. tuberculosis* (MTB), based on the correlation of phenotypic resistance with mutations found in some 150,000 genome-sequenced MTB isolates (20). For the MABC, there are currently nearly 7,000 publicly available genome sequences, 78% of which are from Europe and the Americas (our unpublished data), leaving a significant lack of MABC genomic data from Asia and especially from China. In this study, we used next-generation sequencing to genome sequence the MABC isolates from 81 patients with pulmonary infections diagnosed in Beijing, China, and then correlated the presence of known drug resistance mutations with their phenotypic drug resistance profiles.

## RESULTS

### Collection of MABC clinical isolates

A total of 81 MABC strains were isolated from the sputum samples of 81 patients at the Beijing Chest Hospital between December 2022 and May 2024. The patients included 32 males and 49 females with ages ranging from 16 to 92 years. Among them, 49 had a history of tuberculosis, and 30 had been diagnosed with bronchiectasis (Table S1). The MeltPro Myco Mycobacterium Identification Kit identified 57 strains (70.4%) as *M. abscessu*s and 24 (29.6%) as *M. massiliense*.

### Whole genome sequencing (WGS) analysis

**Fig 1:**
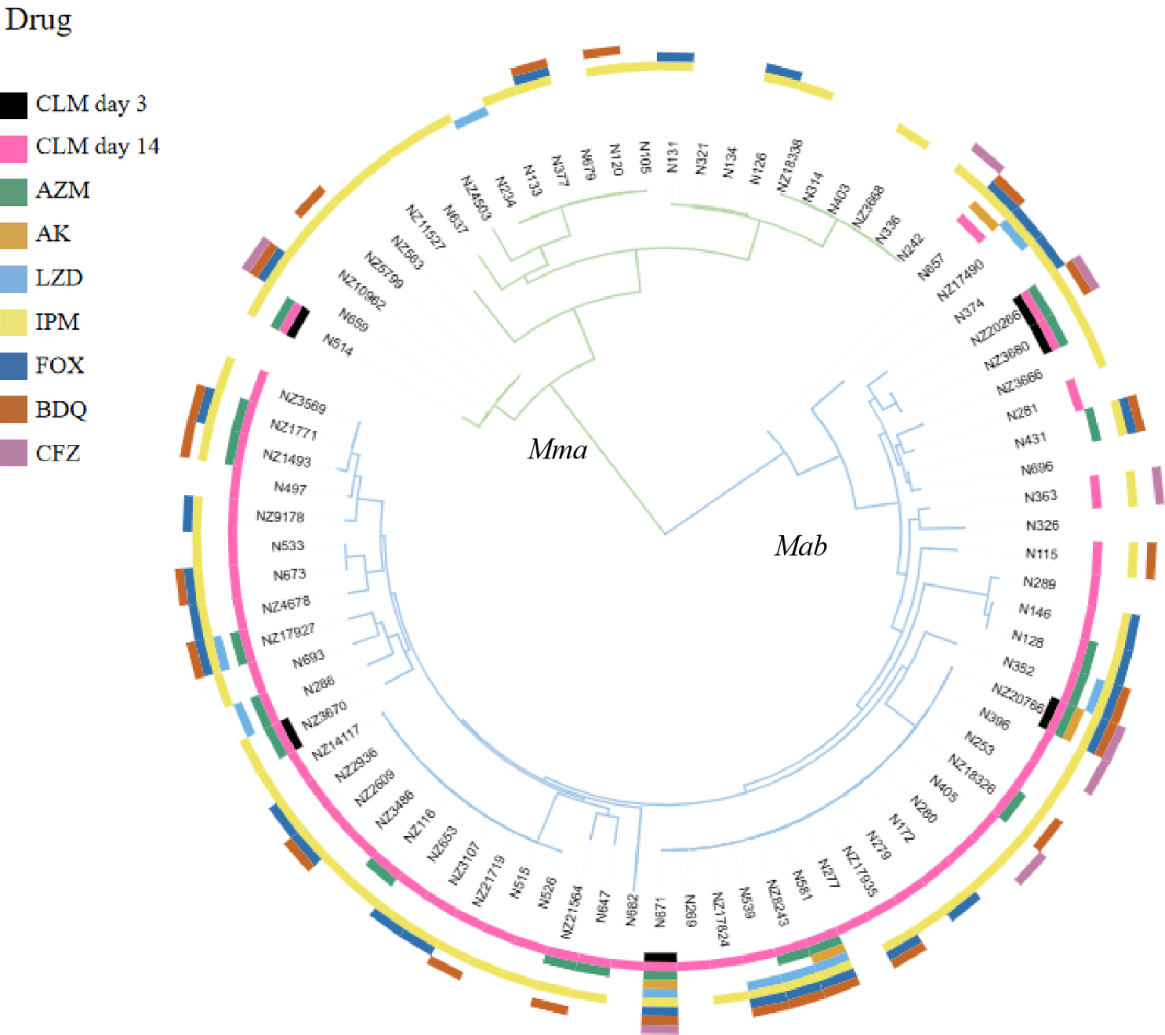
Phylogenetic tree of 81 *MABC* isolates with antibiotic resistance indicated by colors in the outer ring.

We reconstructed a phylogenetic tree using the cumulative total of 225,909 SNPs identified in all 81 isolates (Fig. 1). FastBaps cluster analysis (21) revealed four major genomic clusters that corresponded to clades previously described as dominant circulating clones (DCCs) (22). The largest cluster, DCC1, contained 16 strains, accounting for 19.8% (16/81) of the total isolates.

### Phenotypic antimicrobial susceptibility results

**Fig 2:**
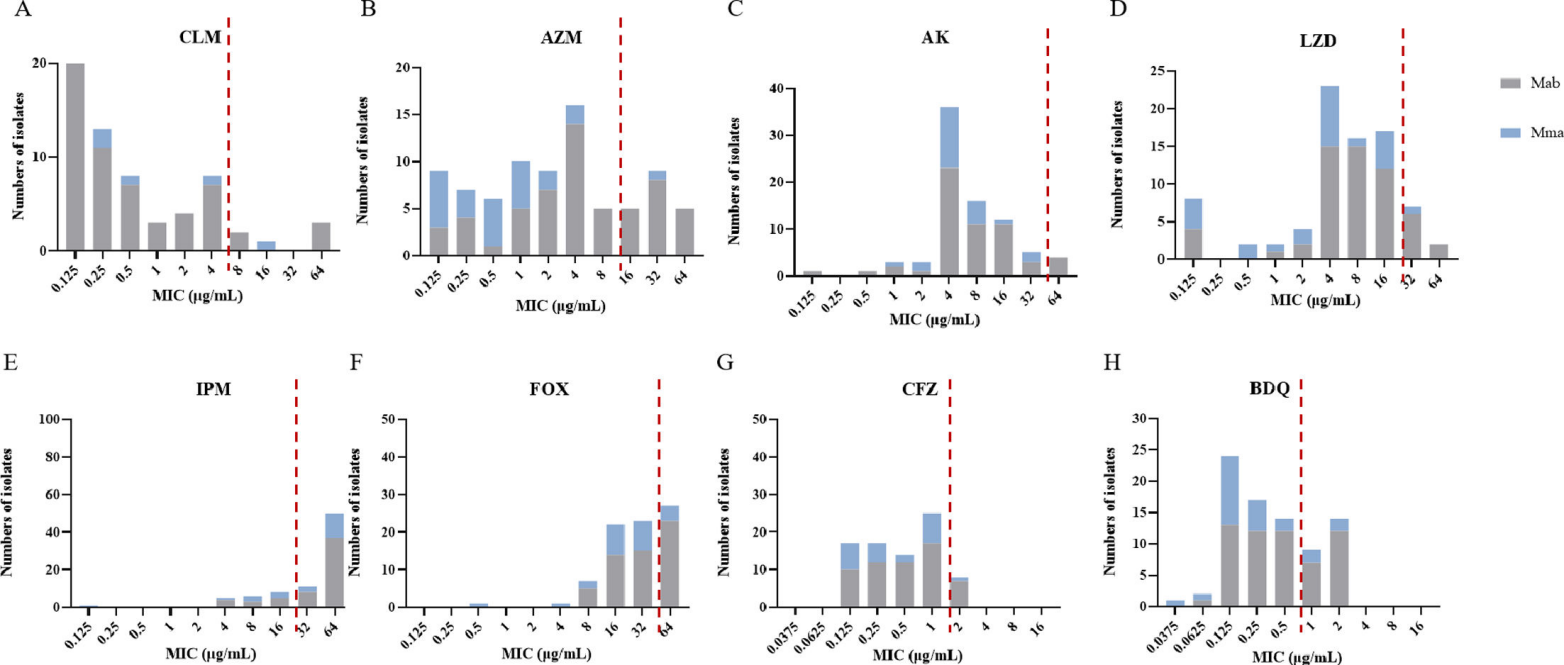
Distribution of MICs to eight antibiotics in 81 *MABC* clinical isolates. MICs to the right of the dotted red lines indicate the cutoff values, such that MICs to the right of these lines are defined as resistance. (**A**) Clarithromycin (CLM), cutoff >8 µg/mL. (**B**) Azithromycin (AZM), cutoff >16 µg/mL. (**C**) Linezolid (LZD), cutoff >32 µg/mL. (**D**) Amikacin (AK), cutoff >64 µg/mL. (**E**) Imipenem (IPM), cutoff >1 µg/mL. (**F**) Cefoxitin (FOX), cutoff >64 µg/mL. (**G**) Bedaquiline, cutoff >0.5 µg/mL. (**H**) Clofazimine, cutoff >1 µg/mL.

MIC testing for eight antibiotics was performed on all 81 MABC strains according to the protocols described in the CLSI M62 (23). By the third day of incubation, turbidity in broth cultures showed that 75.3% (61/81) of strains were resistant to IPM, 33.3% (27/81) were resistant to FOX, 28.4% (23/81) were resistant to BDQ, 11/1% (9/81) were resistant to LZD, 9.9% (8/81) were resistant to CFZ, and 4.9% (4/81) were resistant to AK. Although both CLM and AZM are macrolides, after three days of incubation, 19 strains (23.5%, 19/81) were resistant to AZM, but only six strains (7.4%, 6/81) were resistant to CLM (Fig. 2). To determine the presence of inducible CLM resistance, we extended the observation period to 14 days, during which another 46 strains developed CLM resistance, thereby raising the percentage of CLM resistant strains from 7.4% to 64.2% (52/81) (Table 1). Notably, there was an increase in the CLM MICs from day 3 to day 14 in 49 (94%, 49/52) of the CLM-resistant strains, including three of the six strains that were CLM resistant by day 3. All 49 were *M. abscessus* strains harboring the *erm(41)28T* (*MAB_2297*) mutation associated with inducible CLM resistance. The other three strains that were CLM resistant by day 3 showed no increase in resistance to CLM by day 14 (Table 2). One was an *M. massiliense* strain carrying the *rrl* 2270 (*E. coli* 2058) A-to-G mutation, and the other two were *M. abscessus* strains containing the *erm(41) 28C* mutation (Table 2). Thus, all 49 strains carrying the *erm(41)28T* mutation were *M. abscessus*, all exhibited an increase in their MICs to CLM from day 3 to day 14, and none had rrl mutations. The predictive rate of inducible CLM resistance in strains with the *erm(41*) (*MAB_2297*) 28T was, therefore, 100.0% (49/49) (Fig. S1A). The resistance mechanism in the two strains with the *erm (41*) 28C mutation is unclear (Fig. S1A).

**TABLE 1:** The number of resistant strains and the presence of known resistance-conferring mutations for eight antibiotics used to treat MABC infections

Antibiotic	No. of strains (% of the total 81 strains)			Target gene	No. of resistant strains with target gene mutations (%)	
	Sensitive	Intermediate	Resistant			
Clarithromycin (day 3)	67 (82.7%)	8 (9.9%)	6 (7.4%)	*rrl 2058/2059 (MAB_r5052*)	1 (16.7%)	

				*erm (41)28T(MAB_2297*)	3 (50.0%)	
Clarithromycin (day 14)	28 (34.6%)	1 (2.5%)	52 (64.2%)	*erm (41)28T(MAB_2297*)	49 (100.0%)	


Amikacin	75 (92.6%)	2 (2.5%)	4 (4.9%)	*rrs (MAB_r5051*)	0	
				*rpsl (MAB_3851 c*)	0	
				*eis1 (MAB_4124*)	0	
				*eis2 (MAB_4532 c*)	0	
Linezolid	55 (67.9%)	17 (21.0%)	9 (11.1%)	*rrl(MAB_r5052*)	0	
				*rplC (MAB_3820 c*)	0	
Clofazimine	73 (90.1%)		8 (9.9%)	*MmpL (MAB_1134 c*)	0	
				*MmpS (MAB_1135 c*)	0	
				*TetR (MAB_2299 c*)	0	
				*MmpL (MAB_2301*)	0	
				*MmpS (MAB_2300*)	0	
				*TetR (MAB_4384)*	0	
Bedaquiline	58 (71.6%)		23 (28.4%)	*MmpL (MAB_1134 c*)	0	
				*MmpS (MAB_1135 c*)	0	
				*TetR (MAB_2299 c*)	0	
				*MmpL (MAB_2301*)	0	
				*MmpS (MAB_2300*)	0	
				*TetR (MAB_4384）*	0	
				*AtpE (MAB_1448）*	0	
Cefoxitin	47 (58.0%)	14 (17.3%)	20 (24.7%)	*bla (MAB_2875）*	0	

Imipenem	12 (14.8%)	8 (9.9%)	61 (75.3%)	*bla (MAB_2875）*	12 (14.8%)	

Azithromycin	57 (70.4%)	5 (6.2%)	19 (23.5%)	-^*a*^	-	


^*a*^ -, No mutations related to azithromycin resistance were detected.

**TABLE 2:** Changes in the CLM MICs of MABC isolates from day 3 to day 14 of incubation in CAMHB broth containing different concentrations of CLM^*e*^

Subspecies	Fold change of MIC	Change of MIC (μg/mL)	Resistance shift^*d*^	Number of strains (%)
*M. masilliense*	1	–^*f*^ 16→16	S	15 (18.5%) 1 (1.2%)
			R	
*M. abscessus*		64→64 64→64	R	2 (2.5%)^*b*^ 1 (1.2%)^*c*^
			R	
*M. masilliense*	≤4	0.125→0.25	S	6 (7.4%)^*a*^
*M. abscessus*		0.25→0.5	S	1 (1.2%)^*b*^
		0.5→1	S	1 (1.2%)^*b*^
		0.125→0.5	S	1 (1.2%)^*b*^
		0.125→0.25	S	2 (2.5%)^*b*^
		4→16	I to R	5 (6.2%)^*c*^
*M. masilliense*	≥8	0.125→1	S	1 (1.2%)^*a*^
*M. abscessus*		0.125→1	S	1 (1.2%)^*b*^
		0.125→2	S	1 (1.2%)^*b*^
		4→32	I to R	2 (2.5%)^*c*^
		2→16	S to R	3 (3.7%)^*c*^
		1→8	S to R	1 (1.2%)^*c*^
		1→16	S to R	1 (1.2%)^*c*^
		2→32	S to R	1 (1.2%)^*c*^
		0.25→8	S to R	3 (3.7%)^*c*^
		0.5→16	S to R	6 (7.4%)^*c*^
		1→32	S to R	1 (1.2%)^*c*^
		0.25→16	S to R	6 (7.4%)^*c*^
		0.125→16	S to R	10 (12.3%)^*c*^
		0.25→32	S to R	1 (1.2%)^*c*^
		0.125→32	S to R	6 (7.4%)^*c*^
		8→64	R	2 (2.5%)^*c*^

^*a*^ erm (41) unmutated.

^*b*^ erm (41) 28C.

^*c*^ erm (41) 28T.

^*d*^ S, sensitive; I, intermediate resistance; R, resistant.

^*e*^ CLM resistance was defined as MIC ≥ 8 µg/mL; intermediate resistance as MIC = 4 µg/mL; and susceptible as MIC ≤2 µg/mL.

^*f*^ –, Fifteen strains of *Mycobacterium massiliense* with varying MICs exhibited no changes.

### Limited predictive power of known resistance mutations for other antibiotics

We then sought to correlate known resistance mutations with phenotypic resistance to the other antibiotics, analyzing only those strains with MIC values above the breakpoint for defining resistance, and found that the predictive power was surprisingly poor.

There were four strains with AK resistance, but none had mutations in genes known to be associated with AK resistance: *rrs*, *rpsL*, *eis1*, or *eis2* (Table 1). Similarly, in the nine strains resistant to LZD, the eight strains resistant to CFZ, the 23 strains resistant to BDQ, the 20 strains resistant to FOX, and the 19 strains resistant to AZM (Table 1; Table S6), we detected no mutations associated with resistance to these antibiotics. Of the 61 strains resistant to IPM, we identified *bla* gene mutations in just 12 (19.6%, 12/61): V15A in 10 strains and T116N in two strains, one of which also had a T268A mutation (Fig. S1B).

## DISCUSSION

In this study, we analyzed the WGS data of 81 clinical MABC strains and found that detecting known resistance-related mutations has limited power to predict phenotypic resistance to antibiotics commonly used against MABC infections. Apart from the *rrl* and *erm (41*) genes, which demonstrated a fairly accurate ability to predict CLM resistance, the known mutations associated with resistance to the other antibiotics were not detected in the MABC strains that were phenotypically resistant to these drugs. This finding suggests that other, yet unidentified genes or mechanisms may contribute to MABC resistance.

Notably, even for CLM, a widely studied antibiotic, we found that the *rrl* gene mutation had less-than-ideal predictive power for the isolates that displayed resistance on day 3. This issue has been raised in previous studies from Europe and North America, where approximately 60% to 100% of MABC isolates with acquired CLM resistance harbored *rrl* gene mutations (Table S3). However, in several studies conducted in Asia, the predictive rate was significantly lower, with most studies finding *rrl* mutations in less than 18.5% of strains showing CLM resistance after three days (19). In our study, *rrl* mutations were found in one of the six strains (16.7%, 1/6) with CLM resistance by day 3. One possible reason is that the patients in our cohort were not treatment-naive, and prior use of CLM may have activated the inducible resistance pathway mediated by *erm (41*) T28. This result is consistent with a previous study in China (19) suggesting that the most acquired CLM resistance is actually induced resistance due to *erm (41*) T28 strains. It is also noteworthy that two isolates that developed resistance by day 3 contained the *erm (41) 28C* mutation, which is thought to render this 23S rRNA methyltransferase nonfunctional, suggesting the involvement of additional resistance mechanisms.

Another possible explanation for the lower percentage of *rrl* mutations in Asian strains is that many *M. abscessus* pulmonary infections in Europe and North America occur in patients with cystic fibrosis (CF), whereas CF is relatively uncommon in Asia. This difference in patient background may lead to distinct selection pressures. Our review of previous studies revealed that CLM resistance rates in Europe (average 66.6%, range 56.5%–74.2%) are significantly higher than in Asia (average 23.2%, range 6.8%–45.8%) (15, 16) (Table S2). It remains unknown whether underlying respiratory conditions (e.g., CF and COPD) or ethnic background can influence the development of drug resistance in MABC (24).

The limits of this study include its relatively small sample size of only 81 isolates and the lack of clinical follow-up on treatments and outcomes. In addition, we focused only on the explanatory power of known drug-resistance genes and did not look for novel mutations associated with horizontal gene transfer, although this would likely require a much larger data set. According to the research conducted by Brown-Elliott (25), horizontal gene transfer (HGT) is a significant factor contributing to antimicrobial resistance in some rapidly growing nontuberculous mycobacteria (NTMs). We used next-generation sequencing (NGS) technology to sequence the MABC genomes and then analyzed the sequence data for known resistance-conferring mutations. The short reads generated by NGS make it difficult to identify resistance determinants that are within plasmids or transposons, which often have repetitive sequences. This would be easier with sequencing methods that generate longer reads. This limitation has hindered our ability to thoroughly explore the phenomenon of HGT within the scope of the present study. Herein, visual inspection was employed for result evaluation. However, this method remains inherently subjective, even with the inclusion of a control group. In subsequent investigations, we will employ instrument-based measurements to improve the precision and objectivity of our experimental outcomes. Also, many of the known resistance-conferring mutations were initially identified in *M. tuberculosis*, which is phylogenetically distant from MABC, and therefore, some resistance mechanisms may differ. Additional studies using comparative transcriptional analysis or drug uptake might reveal other mechanisms of antibiotic resistance.

In conclusion, this study systematically analyzed the correlation between known resistance mutations and drug sensitivity phenotypes in 81 clinical MABC isolates by comparing whole-genome sequencing with phenotypic analysis. Apart from CLM resistance, most phenotypic resistance to other antibiotics was not associated with the presence of known resistance mutations, suggesting that other, yet unidentified, mechanisms confer resistance to these antibiotics in MABC.

## MATERIALS AND METHODS

### Collection of MABC clinical isolates

The MABC strains were isolated between December 2022 and May 2024 from sputum samples of patients with pulmonary disease at the Beijing Chest Hospital in Beijing, China. MABC pulmonary disease was managed based on diagnostic and treatment guidelines (26). MABC strains were identified using the MeltPro Myco Mycobacterium Identification Reagent Kit (27) and stored at −80°C in the National Tuberculosis Clinical Laboratory of the Beijing Chest Hospital, which is a reference center for treating TB and NTM.

### Minimum inhibitory concentrations (MICs) testing

For each of the 81 MABC isolates, the MICs were determined for the following antibiotics: CLM, AZM, AK, LZD, the β-lactams IPM and FOX, CFZ, and BDQ. For the microplate AlamarBlue assay (MABA) (28), the stock solutions of AK and IPM were dissolved in water, whereas the remaining drugs were prepared in dimethyl sulfoxide (DMSO). For CFZ, the intermittent ultrasonic method was used until the drug was completely dissolved. The final antibiotic concentrations tested were obtained by serial 2-fold dilutions in cation-adjusted Mueller-Hinton broth in 96-well microtiter plates, such that each well had 100 µL of antibiotic-containing media. Cultures were scraped from Lowenstein-Jensen (LJ) solid media into a sterile homogenization vessel filled with physiological saline and were subjected to a high-speed rotary homogenizer to obtain a uniform suspension. After adjusting to McFarland 0.5 with sterile saline, the bacterial suspension was diluted 200-fold in cation-adjusted Mueller-Hinton broth (CAMHB, Becton Dickinson, USA). Subsequently, 100 µL of the diluted inoculum was added to each well of a 96-well plate with the antibiotic-containing media. For CFZ and BDQ (Cat. No., HY-14881, MedChemExpress, USA), the final concentrations ranged from 0.0375 to 16 µg/mL, whereas the remaining six drugs were assayed across concentrations ranging from 0.125 to 64 µg/mL. The plates were incubated at 37°C for three days, then 30 µL of AlamarBlue (BD, USA) was added to a subset of the wells, and the plates were re-incubated at 37°C for an additional 24 hours before assessing color change. Blue color indicates no growth, while pink color indicates mycobacterial growth (29). The MIC was defined as the lowest concentration of drug that inhibited color change from blue to pink. To detect inducible resistance, the incubation time for CLM was extended. AlamarBlue was added to the remaining wells on day 14, and color change was assessed after an additional 24 hours of incubation. Antibiotic susceptibility and resistance breakpoints were interpreted according to Clinical Laboratory Standards Institute (CLSI) M62 (23) and values in the literature, as shown in Table 3 (30, 31). Based on previous studies (32, 33), the *M. abscessus* strain ATCC 19977 was used as the control reference strain for MIC determinations.

**TABLE 3:** Clinical breakpoints, epidemiological cutoff values (in mg/L), and testing ranges for determining MABC susceptibility to the antibiotics in this study

Antibiotic	This study or CLSI	Sensitive	Intermediate	Resistant	Literature calculated ECOFF^*b*^
Clarithromycin					
	This study	≤2	4	≥8	
	CLSI	≤1	4	≥8	
Azithromycin					
	This study	≤4	8	≥16	
	CLSI	-^*a*^	-	-	
Amikacin					
	This study	≤16	32	≥64	
	CLSI	≤16	32	≥64	
Linezolid					
	This study	≤8	16	≥32	
	CLSI	≤8	16	≥32	
Imipenem					
	This study	≤4	8–16	≥32	
	CLSI	≤4	8–16	≥32	
Cefoxitin					
	This study	≤16	32	≥64	
	CLSI	-	-	-	
Clofazimine					
	This study	<1	-	>1	>1 (24, 25)
	CLSI	-	-	-	
Bedaquiline					
	This study	<0.5	-	>0.5	>0.5 (24, 25)
	CLSI	-	-	-	

^*a*^ -, The CLSI does not currently provide MIC breakpoints for some antibiotics related to *Mycobacterium abscessus* complex (MABC).

^*b*^ The column “Literature calculated ECOFF” indicates that the ECOFF values for these two drugs have not been reported in the CLSI. The ECOFF values referenced in this study are derived from literature.

### DNA extraction and sequencing

Genomic DNA from the MABC isolates was extracted using a Qiagen QIAamp DNA Mini Kit, as previously described (34), and subjected to multiplexed paired-end sequencing on the Illumina Hiseq platform.

### Subspecies assignment

We performed *de novo* assemblies of sequencing reads using SPAdes v3.11.1 (35). These contigs were then compared to reference sequences for each of the three subspecies: *subsp. abscessus* GZ002 (NZ_CP034181.1), *subsp. massiliense* CCUG48898 (NZ_AP014547.1), and *subsp. bolletii* GD91 (NZ_CP065265.1). To identify the subspecies assignment of each MAB strain, we calculated a whole-genome-based average nucleotide identity (gANI) score using fastANI v1.2 with default parameters (36). The subspecies assignments were based on average nucleotide identity (gANI) scores of at least 98% compared to the reference strains of the three subspecies.

### Variant calling

Sickle Tool (37) was used to prune sequencing reads, retaining reads with Phred base quality above 20 and a read length greater than 30. BWA MEM v0.7.17 (38) was used to map sequencing reads using the corresponding subspecies reference sequences as templates. SAMtools (version 1.3.1) (37) was used for SNP calling with a mapping quality greater than 3. Fixed (frequency 95%) and unfixed (frequency <95%) mutations, insertion, and deletions were identified with VarScan (version 2.3.9). SNPs in repetitive regions of the genome (PPE/PE-PGRS family genes, phage sequences, insertions, and mobile genetic elements) were excluded.

### Phylogenetic reconstruction

We inferred maximum likelihood (ML) phylogenetic trees for the 81 isolates with FastTree (39) and the General Time-Reversible (GTR) model of nucleotide substitution with four gamma rate categories. To validate the trees, an ML phylogenetic tree was inferred using IQ-TREE v2 (35) with ultrafast bootstrap support from 1,000 replications. The best-fit nucleotide substitution model was GTR+I+G as determined by ModelFinder (40). Phylogeny trees were visualized in FigTree (version 1.4.3) or iTOL (41).

### Known mutations related to drug resistance

WGS data were searched for mutations associated with drug resistance (Table S4) by comparing the extracted base calls from the vcf files to known resistance-conferring (4) alleles using in-house Python scripts.

## Data Availability

All sequence data associated with this study was deposited in the European Nucleotide Archive under project accession PRJNA1182993.

## SUPPLEMENTAL MATERIAL

Supplemental material aac.01847-24-s0001.docx Tables S1 to S5; Fig. S1.

Table S6 aac.01847-24-s0002.xlsx MIC data and genetic analysis of different strains.

ASM does not own the copyrights to Supplemental Material that may be linked to, or accessed through, an article. The authors have granted ASM a non-exclusive, world-wide license to publish the Supplemental Material files. Please contact the corresponding author directly for reuse.
