# Supplementary material for: Limited predictive power of known resistance genes for phenotypic drug resistance in clinical Mycobacterium abscessus complex from Beijing in China
Source: Antimicrob Agents Chemother. 2025 May 27;69(7):e01847-24. doi: 10.1128/aac.01847-24 (PMC12217466; doi:10.1128/aac.01847-24)
Supplement: Supplemental material — Tables S1 to S5; Fig. S1. [file aac.01847-24-s0001.docx]

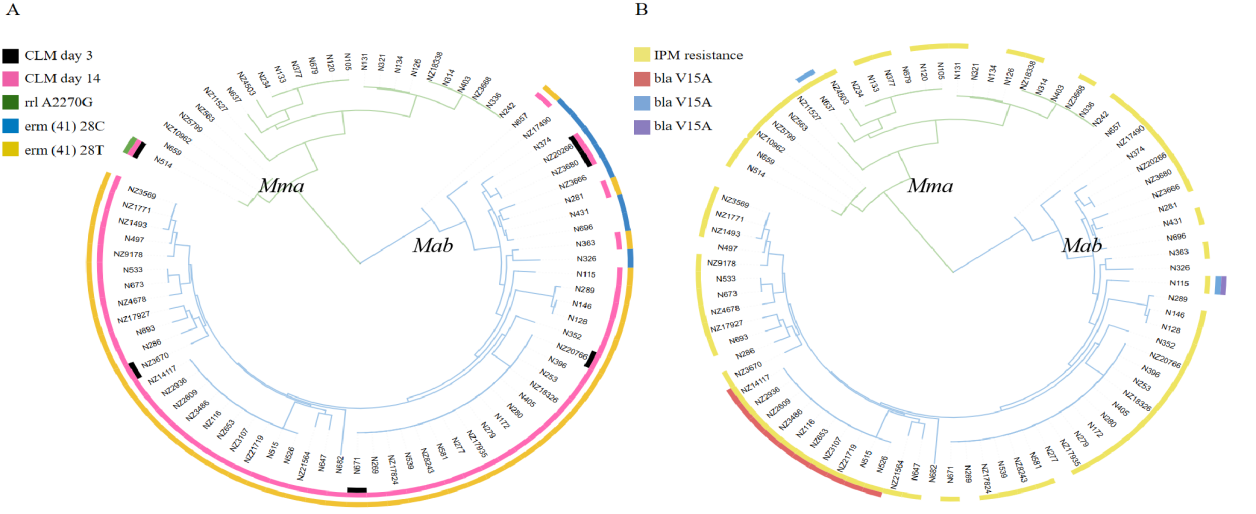

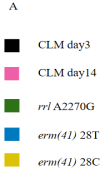

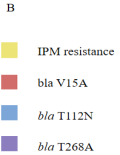


Fig S1 Distribution of drug-resistant mutations of clarithromycin and imipenem. (A) Distribution of mutations in the *rrl* and *erm(41)* genes in clarithromycin. (B) Mutation distribution of *bla* gene in imipenem.

Table S1 Clinical Information on *MABC* Patients

|  | Resistance（65） | Sensitive（16） | *P* value |
| --- | --- | --- | --- |
| **Patient information** |  |  |  |
| **Gender** |  |  |  |
| male | 26 | 6 | 0.855 |
| female | 39 | 10 |  |
| smoking | 12 | 5 | 0.26 |
| tipple | 6 | 3 | 0.521 |
| **Clinical Features** |  |  |  |
| cough | 50 | 15 | 0.244 |
| expectoration | 45 | 14 | 0.247 |
| lose weight | 18 | 8 | 0.087 |
| fever | 25 | 8 | 0.4 |
| hemoptysis | 15 | 2 | 0.557 |
| chest distress | 10 | 1 | 0.584 |
| thoracodynia | 8 | 2 | 1 |
| dyspnea | 2 | 1 | 1 |
| **Complications** |  |  |  |
| bronchiectasis | 21 | 9 | 0.076 |
| hypertension | 8 | 2 | 1 |
| malnutrition | 17 | 4 | 1 |
| Immune diseases | 3 | 1 | 1 |
| diabetes | 3 | 0 | 0.891 |
| chronic gastritis | 8 | 0 | 0.312 |
| **Bacteriological factors** |  |  |  |
| sputum smear | 31 | 8 | 0.869 |
| cultivate | 53 | 10 | 0.101 |
| Subspecies  *M. abscessus*  *M. massiliense* | 48  18 | 9  6 | 0.167 |

Table S2 Drug resistance rates of 8 drugs in different clinical studies

| NATION | YEAR | ISOLATE | CLM | AZI | AMK | LZD | IPM | FOX | BDQ | CFZ | REF |
| --- | --- | --- | --- | --- | --- | --- | --- | --- | --- | --- | --- |
| THIS STUDY | 2024 | 81 | 6  (16.7%) | 19  (23.5%） | 4  (4.9%) | 9  (11.1%) | 61  (75.3%) | 27  (33.3%) | 23  (28.4%) | 8  (9.9%) |  |
| China | 2021 | 43 | 6  （14.0%） | 5  （11.6%） | 1  （2.3%） | 1  （2.3%） | 0 | 6  （14.0%） | - | - | [^1^] |
| China | 2017 | 67 | 18  （26.0%） | - | 4  （6.0%） | 47  （70.1%） | 41  （61.2%） | 14  （20.1%） | - | - | [^2^] |
| Japan | 2020 | 102 | 7  (6.8%) | - | 6  (5.9%) | 11 (10.8%) | 2  (2.0%) | - | - | - | [^3^] |
| Japan | 2018 | 24 | 11  （45.8%） | 11  （45.8%） | 0 | 0 | 0 | - | - | - | [^4^] |
| America | 2019 | 85 | 48  （56.5%） | - | 4  （4.7%） | 72  （84.7%） | 76  （89.4%） | 17  （20%） | - | - | [^5^] |
| Germany | 2017 | 29 | 20  (69%) | - | 1  （3.4%） | 2  （6.9%） |  | 4  （13.8%） | - | - | [^6^] |
| England | 2018 | 62 | 46  (74.2%) | - | 15  (24.5%) | 47  (75.8%） | - | 17  (28.8%) | - | - | [^7^] |
| Australia | 2015 | 42 | 18  （42.9%） | - | 2  （4.8%） | 7  （16.7%） | 26  （61.9%） | 7  （16.7%） | - | - | [^8^] |
| South Korea | 2014 | 404 | 137  （33.9%） | - | 37  （9.2%） | - | - | - | - | - | [^9^] |

Table S3 Distribution and CLM susceptibility patterns among Mycobacterium abscessus genotypes^a^

| Country | Subspecies | Cut-off | Erm(41) | | 3days resistant | rrl mut | rrl mut /3days resistant | Induced resistance^b^ | | 14 days susceptible and intermediate | |
| --- | --- | --- | --- | --- | --- | --- | --- | --- | --- | --- | --- |
|  |  |  | T28 | C28 |  |  |  | T28 | C28 | T28 | C28 |
| China（81） | *M. abscessus(57)* | ≥8 | 49 | 8 | 5 | 0 | 0%（0/5） T28 60%（3/5） | 100% (45/45) | 0 | 0% (0/45) | 75% (6/8) |
|  | *M. massiliense(24)* |  |  |  | 1 | 1 | 100%（1/1） |  |  |  |  |
| China^d^（162） | *M. abscessus(123)* | ≥8 | 108 | 15 | 46 | 3 | 6.5%（3/46） | 100% (62/62) | 0 | 2.3% (1/43) | 32.6% (14/43) |
|  | *M. massiliense(39)* |  |  |  | 9 | 7 | 77.8%(7/9) |  |  |  |  |
| Spain^d^（12） | *M. abscessus(12)* | ≥8 | 8 | 4 | 4 | 4 | 100% (3/3) | 100% (5/5) | 0 | nd^c^ | nd |
|  | *M. massiliense(0)* |  |  |  |  |  |  |  |  |  |  |
| France^d^（140） | *M. abscessus(112)* | ≥16 | 94 | 18 | 10(T28) | 7 | 70% (7/10) | 100% (84/84) | 0 | 0 | 40.9% (18/44) |
|  | *M. massiliense(28)* |  |  |  | 2 | 2 | 100%（2/2） |  |  |  |  |
| US^d^（349） | *M. abscessus* | ≥8 | 287 | 62 |  |  | nd | 100% (264/264) | 0 | 11.1 (7/63) | 88.9% (56/63) |
|  | *M. massiliense* |  |  |  |  |  |  |  |  |  |  |
| China^d^ (133) | *M. abscessus(133)* | ≥8 | 62(20) | 71(39) | 59(20+39) |  | 44.4%(59/133) 32.3%(20/62) 54.9%(39/71) |  |  |  |  |
|  | *M. massiliense(0)* |  |  |  |  |  |  |  |  |  |  |
| England^d^ (167) | *M. abscessus(131)* | ≥8 | 94 | 37 |  | 5 |  |  |  |  |  |
|  | *M.. massiliens(36)* |  | 1 |  |  | 11 |  |  |  |  |  |

^a^Genotypes include *erm(41)C28*, *erm(41)T28*, *M type* and *rrl* 2058/2059 mutation (*rrl* mut).

^b^Sensitive/ intermediate at ERT, but resistant at LRT.

^c^no data

^d^Data obtained from Li B *et al.,*2017and Brown-Elliott *et al.*, 2015; Rubio *et al.*, 2015 and Mougari *et al.*, 2016，Timothy Walkera *et al*.，2021，Tao Luo *et al*.，2024.

Supplementary Table S4 Literature of Mutation associated *M. abscessus* drug resistance

| Antibiotics | Genes | *E.coli* | *M.abscessus* | Remark | Ref |
| --- | --- | --- | --- | --- | --- |
| Amikacin | *rrs* |  | A-33G |  | [^10^] |
|  |  | T1406A | T1372A |  | [^11^] |
|  |  | A1408G | A1374G |  | [^10^, ^11^] |
|  |  | C1409T | C1375T |  | [^11^] |
|  |  | G1491T | G1455T |  | [^11^] |
|  |  | C1496T | C1460T |  | [^12^] |
|  |  | T1498A | T1462A |  | [^12^] |
|  | *rpsl* |  | MAB_3851c |  | [^13^] |
|  | *eis1* |  | MAB_4124 |  | [^14^] |
|  | *eis2* |  | MAB_4532c |  | [^15^] |
| Clarithromycin | *erm(41)* |  | C28T |  | [^9^,^16^,^17^] |
|  |  |  | T19C |  | [^18^] |
|  |  |  | A-31T |  | [^10^] |
|  | *rrl* |  | G795A |  | [^10^] |
|  |  |  | T371C |  | [^10^] |
|  |  |  | T1401C |  | [^10^] |
|  |  |  | A1932G |  | [^10^] |
|  |  |  | A2039C |  | [^10^] |
|  |  | A2057G | A2269G |  | [^10^] |
|  |  | A2058G | A2270G |  | [^19^,^20^] |
|  |  | A2058C | A2270C |  | [^19^,^20^] |
|  |  | A2058T | A2270T |  | [^19^,^20^] |
|  |  | A2059T | A2271T |  | [^19^,^20^] |
|  |  | G2067A | G2279A |  | [^10^] |
|  |  | A2069A | G2281A |  | [^10^] |
|  |  | A2082C | A2293C |  | [^10^] |
| Linezolid | *rplC (L3)* |  | T54A |  | [^21^] |
|  | *rplD (L4)* |  | G142A |  | [^21^] |
|  |  |  | A175P |  | [^21^] |
|  | *rrl (23S rRNA)* |  | G15A | Only R strain | [^21^] |
|  |  |  | T328C | Only R strain | [^21^] |
|  |  |  | G348A | Only R strain | [^21^] |
|  |  |  | C1445T | Only R strain | [^21^] |
|  |  |  | C1582A | Only R strain | [^21^] |
|  |  |  | T2138C | Only R strain | [^21^] |
|  |  |  | A2271C | Only R strain | [^21^] |
|  |  |  | C2432T | Only R strain | [^21^] |
|  |  |  | G3048A | Only R strain | [^21^] |
|  |  |  | T54A | R+S strain | [^21^] |
|  |  |  | C109T | R+S strain | [^21^] |
|  |  |  | G399A | R+S strain | [^21^] |
|  |  |  | A437G | R+S strain | [^21^] |
|  |  |  | C633T | R+S strain | [^21^] |
|  |  |  | C742T | R+S strain | [^21^] |
|  |  |  | A1717G | R+S strain | [^21^] |
|  |  |  | A2270C | R+S strain | [^21^] |
|  |  |  | T3001C | R+S strain | [^21^] |
|  |  |  | C3042T | R+S strain | [^21^] |
| Bedaquiline and Clofazimine | *mmpL* |  | MAB 4382c | in vitro induced resistance | [^22^] |
|  | *mmpS* |  | MAB 4383c | in vitro induced resistance | [^22^] |
|  | *tetR* |  | MAB 4384 | in vitro induced resistance | [^22^,^23^] |
|  | *mmpL* |  | MAB 1134c | in vitro induced resistance | [^24^] |
|  | *mmpS* |  | MAB 1135c | in vitro induced resistance | [^24^] |
|  | *tetR* |  | MAB 2299c | in vitro induced resistance | [^24^] |
|  | *mmpS* |  | MAB 2300 | in vitro induced resistance | [^24^] |
|  | *mmpL* |  | MAB 2301 | in vitro induced resistance | [^24^] |
| Bedaquiline | *AtpE* |  | （MAB_1448） |  | [^25^] |
| Cefoxitin and  Imipenem | *bla* |  | （MAB_2875） |  | [^26^] |

Table S5 Drug Source and Catalog Number

| Drug | Catalog Number | Manufacturer | Solvent |
| --- | --- | --- | --- |
| Clarithromycin | HY-17508 | MCE (MedChemExpress) Company | DMSO |
| Azithromycin | HY-17506 | MCE (MedChemExpress) Company | DMSO |
| Amikacin | HY-B0509A | MCE (MedChemExpress) Company | H2O |
| Linezolid | HY-10394 | MCE (MedChemExpress) Company | DMSO |
| Imipenem | HY-B1369A | MCE (MedChemExpress) Company | H2O |
| Cefoxitin | HY-B1825 | MCE (MedChemExpress) Company | DMSO |
| Clofazimine | HY-B1046 | MCE (MedChemExpress) Company | DMSO |
| Bedaquiline | HY-14881 | MCE (MedChemExpress) Company | DMSO |

**Reference:**

1. Guo, Q. *et al.* Antimicrobial susceptibility profiles of Mycobacterium abscessus complex isolates from respiratory specimens in Shanghai, China. *Journal of Global Antimicrobial Resistance* **25**, 72–76 (2021).

2. Lee, M.-C. *et al.* Antimicrobial resistance in Mycobacterium abscessus complex isolated from patients with skin and soft tissue infections at a tertiary teaching hospital in Taiwan. *Journal of Antimicrobial Chemotherapy* **72**, 2782–2786 (2017).

3. Shirata, M., Yoshimoto, Y., Marumo, S., Tamai, K. & Fukui, M. In vitro efficacy of combinations of eight antimicrobial agents against Mycobacteroides abscessus complex. *International Journal of Infectious Diseases* **97**, 270–277 (2020).

4. Kusuki, M. *et al.* Determination of the antimicrobial susceptibility and molecular profile of clarithromycin resistance in the Mycobacterium abscessus complex in Japan by variable number tandem repeat analysis. *Diagnostic Microbiology and Infectious Disease* **91**, 256–259 (2018).

5. Aono, A. *et al.* Antimicrobial susceptibility testing of Mycobacteroides (Mycobacterium) abscessus complex, Mycolicibacterium (Mycobacterium) fortuitum, and Mycobacteroides (Mycobacterium) chelonae. *Journal of Infection and Chemotherapy* **25**, 117–123 (2019).

6. Ruedas-López, A. *et al.* Subspecies Distribution and Antimicrobial Susceptibility Testing of Mycobacterium abscessus Clinical Isolates in Madrid, Spain: a Retrospective Multicenter Study. *Microbiol Spectr* **11**, e05041-22 (2023).

7. Cowman, S., Burns, K., Benson, S., Wilson, R. & Loebinger, M. R. The antimicrobial susceptibility of non-tuberculous mycobacteria. *Journal of Infection* **72**, 324–331 (2016).

8. Chua, K. Y. L., Bustamante, A., Jelfs, P., Chen, S. C.-A. & Sintchenko, V. Antibiotic susceptibility of diverse Mycobacterium abscessus complex strains in New South Wales, Australia. *Pathology* **47**, 678–682 (2015).

9. Lee, S. H. *et al.* The Drug Resistance Profile of *Mycobacterium abscessus* Group Strains from Korea. *Ann Lab Med* **34**, 31–37 (2014).

10. Bryant, J. M. *et al.* Whole-genome sequencing to identify transmission of Mycobacterium abscessus between patients with cystic fibrosis: a retrospective cohort study. *The Lancet* **381**, 1551–1560 (2013).

11. Nessar, R., Reyrat, J. M., Murray, A. & Gicquel, B. Genetic analysis of new 16S rRNA mutations conferring aminoglycoside resistance in Mycobacterium abscessus. *Journal of Antimicrobial Chemotherapy* **66**, 1719–1724 (2011).

12. Kim, S.-Y. *et al.* Association between 16S rRNA gene mutations and susceptibility to amikacin in Mycobacterium avium Complex and Mycobacterium abscessus clinical isolates. *Sci Rep* **11**, 6108 (2021).

13. Dal Molin, M. *et al.* Molecular Mechanisms of Intrinsic Streptomycin Resistance in Mycobacterium abscessus. *Antimicrob Agents Chemother* **62**, e01427-17 (2018).

14. Rominski, A. *et al.* Elucidation of Mycobacterium abscessus aminoglycoside and capreomycin resistance by targeted deletion of three putative resistance genes. *Journal of Antimicrobial Chemotherapy* **72**, 2191–2200 (2017).

15. Ung, K. L., Alsarraf, H. M. A. B., Olieric, V., Kremer, L. & Blaise, M. Crystal structure of the aminoglycosides *N* ‐acetyltransferase Eis2 from *Mycobacterium abscessus*. *The FEBS Journal* **286**, 4342–4355 (2019).

16. Rubio, M. *et al.* Inducible and Acquired Clarithromycin Resistance in the Mycobacterium abscessus Complex. *PLoS ONE* **10**, e0140166 (2015).

17. a novel gene erm 41 confers induc source antimicrob agents chemother so 2009 apr 53 4 1367 76.pdf.pdf.

18. Kim, S.-Y., Shin, S. J., Jeong, B.-H. & Koh, W.-J. Successful antibiotic treatment of pulmonary disease caused by Mycobacterium abscessus subsp. abscessus with C-to-T mutation at position 19 in erm(41) gene: case report. *BMC Infect Dis* **16**, 207 (2016).

19. Wallace, R. J. *et al.* Genetic basis for clarithromycin resistance among isolates of Mycobacterium chelonae and Mycobacterium abscessus. *Antimicrob Agents Chemother* **40**, 1676–1681 (1996).

20. Pfister, P. *et al.* The Structural Basis of Macrolide–Ribosome Binding Assessed Using Mutagenesis of 23S rRNA Positions 2058 and 2059. *Journal of Molecular Biology* **342**, 1569–1581 (2004).

21. Kim, S.-Y. *et al.* Genetic mutations in linezolid-resistant Mycobacterium avium complex and Mycobacterium abscessus clinical isolates. *Diagnostic Microbiology and Infectious Disease* **94**, 38–40 (2019).

22. Halloum, I. *et al.* Resistance to Thiacetazone Derivatives Active against Mycobacterium abscessus Involves Mutations in the MmpL5 Transcriptional Repressor MAB_4384. *Antimicrob Agents Chemother* **61**, e02509-16 (2017).

23. Richard, M. *et al.* Mechanistic and Structural Insights Into the Unique TetR-Dependent Regulation of a Drug Efflux Pump in Mycobacterium abscessus. *Front. Microbiol.* **9**, 649 (2018).

24. Gutiérrez, A. V., Richard, M., Roquet-Banères, F., Viljoen, A. & Kremer, L. The TetR Family Transcription Factor MAB_2299c Regulates the Expression of Two Distinct MmpS-MmpL Efflux Pumps Involved in Cross-Resistance to Clofazimine and Bedaquiline in Mycobacterium abscessus. *Antimicrob Agents Chemother* **63**, e01000-19 (2019).

25. Calvet-Seral, J. *et al.* Targeted Chromosomal Barcoding Establishes Direct Genotype-Phenotype Associations for Antibiotic Resistance in Mycobacterium abscessus. *Microbiol Spectr* **11**, e05344-22 (2023).

26. Soroka, D. *et al.* Characterization of broad-spectrum Mycobacterium abscessus class A -lactamase. *Journal of Antimicrobial Chemotherapy* **69**, 691–696 (2014).
